# Sublethal hyperthermia enhances anticancer activity of doxorubicin in chronically hypoxic HepG2 cells through ROS-dependent mechanism

**DOI:** 10.1042/BSR20210442

**Published:** 2021-06-11

**Authors:** Qi Wang, Hui Zhang, Qian-qian Ren, Tian-he Ye, Yi-ming Liu, Chuan-sheng Zheng, Guo-feng Zhou, Xiang-wen Xia

**Affiliations:** 1 Department of Radiology, Union Hospital, Tongji Medical College, Huazhong University of Science and Technology, 1277 Jiefang Avenue, Wuhan City, Hubei Province 430022, China; 2 Key laboratory of Molecular Medical Imaging, Hubei Province, 1277 Jiefang Avenue, Wuhan City, Hubei Province 430022, China; 3 Department of Internal Medicine, Wuhan Hankou Hospital, 172 Zhaojiatiao road, Wuhan City, Hubei Province 430011, China

**Keywords:** doxorubicin, HepG2, hyperthermia, hypoxia, reactive oxygen species, redox equilibrium

## Abstract

Thermal ablation in combination with transarterial chemoembolization (TACE) has been reported to exert a more powerful antitumor effect than thermal ablation alone in hepatocellular carcinoma patients. However, the underlying mechanisms remain unclear. The purpose of the present study was to evaluate whether sublethal hyperthermia encountered in the periablation zone during thermal ablation enhances the anticancer activity of doxorubicin in chronically hypoxic (encountered in the tumor area after TACE) liver cancer cells and to explore the underlying mechanisms. In the present study, HepG2 cells precultured under chronic hypoxic conditions (1% oxygen) were treated in a 42°C water bath for 15 or 30 min, followed by incubation with doxorubicin. Assays were then performed to determine intracellular uptake of doxorubicin, cell viability, apoptosis, cell cycle, mitochondrial membrane potential (MMP), reactive oxygen species (ROS), and total antioxidant capacity. The results confirmed that sublethal hyperthermia enhanced the intracellular uptake of doxorubicin into hypoxic HepG2 cells. Hyperthermia combined with doxorubicin led to a greater inhibition of cell viability and increased apoptosis in hypoxic HepG2 cells as compared with hyperthermia or doxorubicin alone. In addition, the combination induced apoptosis by increasing ROS and causing disruption of MMP. Pretreatment with the ROS scavenger N-acetyl cysteine significantly inhibited the apoptotic response, suggesting that cell death is ROS-dependent. These findings suggested that sublethal hyperthermia enhances the anticancer activity of doxorubicin in hypoxic HepG2 cells via a ROS-dependent mechanism.

## Introduction

Hepatocellular carcinoma (HCC) is one of the most common malignancies worldwide [1,2]. Thermal ablation, predominantly radiofrequency ablation (RFA) and microwave ablation (MWA), has shown excellent therapeutic efficacy in HCC and is a commonly used therapy following surgery and embolotherapy (transarterial chemoembolization/transarterial embolization, TACE/TAE) [3–5]. Several studies have shown that RFA can achieve complete necrosis in most lesions smaller than 3 cm in diameter [6,7]. However, for lesions larger than 3 cm in diameter, especially for those over 5 cm, complete necrosis cannot be obtained in a minority of patients, even if the procedure is repeated. Clinical studies have reported that residual viable tumor cells have been identified in up to 20% of clinical cases following ablation [8,9].

Several studies have shown that TACE in combination with RFA increases the size of the ablation zone as compared with RFA treatment alone, and the efficacy has been demonstrated in the treatment of small and medium-sized HCC [10–13]. The mechanisms underlying the potential advantage of combined therapies remain unclear. One competitive theory is that hyperthermia during thermal ablation may increase the sensitivity of tumor cells to cytotoxicity from chemotherapeutic agents, such as doxorubicin [14,15]. However, the underlying mechanisms have not been sufficiently investigated, and further studies are needed.

It is well known that during thermal ablation, the temperature inside the ablation zone decreases from the center to the margin, where the temperature is sublethal and residual viable tumor cells often exist, which potential cause tumor relapse [16,17]. In our previous study, we reported that chronic hypoxia, an important characteristic following TACE, triggers chemoresistance to doxorubicin in HepG2 cells [18]. In the present study, we used *in vitro* methods to investigate whether sublethal hyperthermia, which is usually encountered in the periablation zone during thermal ablation, promotes the sensitivity of hypoxic HepG2 cells to cytotoxicity from doxorubicin.

## Materials and methods

### Cell culture

Human liver cancer-derived HepG2 cells were purchased from the China Center for Type Culture Collection (Wuhan University, Hubei, China) and were authenticated by short tandem repeat (STR) profiling according to the American Type Culture Collection (ATCC) guidelines. Cells were grown in a monolayer in tissue culture flasks (Corning, NY, U.S.A.) in RPMI-1640 medium (Thermo Fisher, MA, U.S.A.) containing 10% fetal bovine serum (Thermo Fisher, MA, U.S.A.), 1% sodium pyruvate (Caisson Labs, UT, U.S.A.), 1% nonessential amino acids (Thermo Fisher, MA, U.S.A.), and 1 μM gentamicin (Thermo Fisher, MA, U.S.A.) at 37°C in a humidified incubator with 5% carbon dioxide. Cells in logarithmic growth phase were used.

The chronic hypoxia condition was achieved as described previously [18]. Briefly, cells were subjected repeatedly to hypoxia (1% O_2_, 5% CO_2_, 94% N_2_) for 4 h daily for seven consecutive days. The following treatments and assays were performed under normoxic conditions.

### Heat treatments

Confluent cells in six-well culture plates containing 2 ml of medium per well were heated for 15 or 30 min at 42°C in temperature-controlled precision water baths (± 0.1°C) (Haake D8, Fisher Scientific, Montreal, QC). Cells were then cultured in fresh medium at 37°C in the presence (1 μM, working concentration) or absence of doxorubicin for various lengths of time until the following assays were performed (3 h for intracellular uptake of doxorubicin; 24 h for apoptosis, cell cycle, reactive oxygen species, and total antioxidant capacity assays; 2 h for mitochondrial membrane potential) (Supplementary Figure S1).

### Intracellular uptake of doxorubicin

After heating as described above, cells were then incubated in fresh medium without doxorubicin for 3 h. Intracellular doxorubicin was then measured by fluorescence intensity using a flow cytometer (BD Biosciences, CA, U.S.A.) at 488 nm. Data were analyzed using FlowJo X software.

### Clonogenic survival assay

A cell clonogenic survival assay was performed as previously described [19]. In brief, 200 chronic hypoxic HepG2 cells were seeded per well in six-well plates containing 2 ml of complete medium. After 4 h of incubation at 37°C, cells were heated as described previously, followed by incubation with or without doxorubicin for 24 h. Cells were then washed with PBS and allowed to grow for 7 days in complete medium at 37°C and 5% of CO_2_. The visible colonies were fixed with methanol/acetic acid/water (1:1:8 v/v/v) and stained with Giemsa 5% (v/v) in Sorensen phosphate buffer (pH 6.8). The colonies were counted, and the cell survival fraction was determined relative to the untreated control.

### Cell cycle analysis

Cell cycle analysis was performed according to the manufacturer’s manual. In brief, cells were fixed in 70% ice-cold ethanol and stored at -20°C overnight. After washing with PBS, cells were incubated with 300 μl PBS, 125 μl RNase, and 25 μl PI (1 mg/ml) for 30 min at 37°C. A FACSCalibur flow cytometer (BD Bioscience, Heidelberg, Germany) was used to record 10,000 ungated events for each sample at an excitation of 535 nm. Analysis of the events was performed using Modfit 3.2 software.

### Apoptosis

An apoptosis assay was performed using a FITC Annexin V/Dead Cell Apoptosis Kit (Invitrogen), according to the manufacturer’s manual. In brief, cells were harvested and washed twice with cold PBS. The cells were then resuspended in 100 μl of 1× Annexin V Binding Buffer, supplemented with 5 μl of Annexin V-FITC and 1 μl of propidium iodide, and incubated on ice in the dark for 15 min. Thereafter, 400 μl of 1× Annexin V Binding Buffer was added to the sample, and the stained cells were immediately analyzed by a flow cytometer (BD Biosciences). Data were analyzed using FlowJo X software. Annexin V^+^/PI^−^ cells were considered apoptotic and were analyzed as a percentage of the entire cell population.

### Mitochondrial membrane potential (MMP)

MMP was detected using the tetramethylrhodamine methyl ester (TMRM) MMP assay kit, according to the manufacturer’s manual. Cells were labeled with 10 nmol/l tetramethylrhodamine methyl ester (TMRM) in serum-free MEM medium for 20 min at 37°C. Samples were analyzed by flow cytometry. Data were analyzed using FlowJo X software.

### Measurement of reactive oxygen species (ROS)

Cellular ROS levels, specifically hydrogen peroxide levels, were detected using an ROS assay kit, according the manufacturer’s manual. Briefly, cells were incubated with DCFH-DA at a final concentration of 25 μM at 37°C for 30 min. To measure ROS generation, a FACSCalibur flow cytometer (BD Bioscience, Heidelberg, Germany) was used to detect the fluorescence intensity of dichlorofluorescein (DCF) at 535 nm. For each analysis, 10,000 events were recorded.

### Total antioxidant capacity assay

The cellular total antioxidant capacity (TAC) was quantified using a TAC assay kit (Biovision, Mountain View, CA), according to the manufacturer’s instructions. The concentration of the TAC was calculated from the standard curves, and the value was expressed as nmol/ng protein.

### Statistical analysis

All of the experiments were repeated independently three times. The mean ± SD of the three experiments are presented. Data were subjected to Shapiro–Wilk test of normality. Data with normal distribution were analyzed by a one-way ANOVA plus a multiple comparison test (Fisher’s protected least significant difference test, PLSD), and those with abnormal distribution were analyzed via a Kruskal–Wallis test. *P*-values < 0.05 were regarded as significant for all of the statistical analyses.

## Results

### Enhanced cytotoxicity of doxorubicin following sublethal hyperthermia

**Figure 1:**
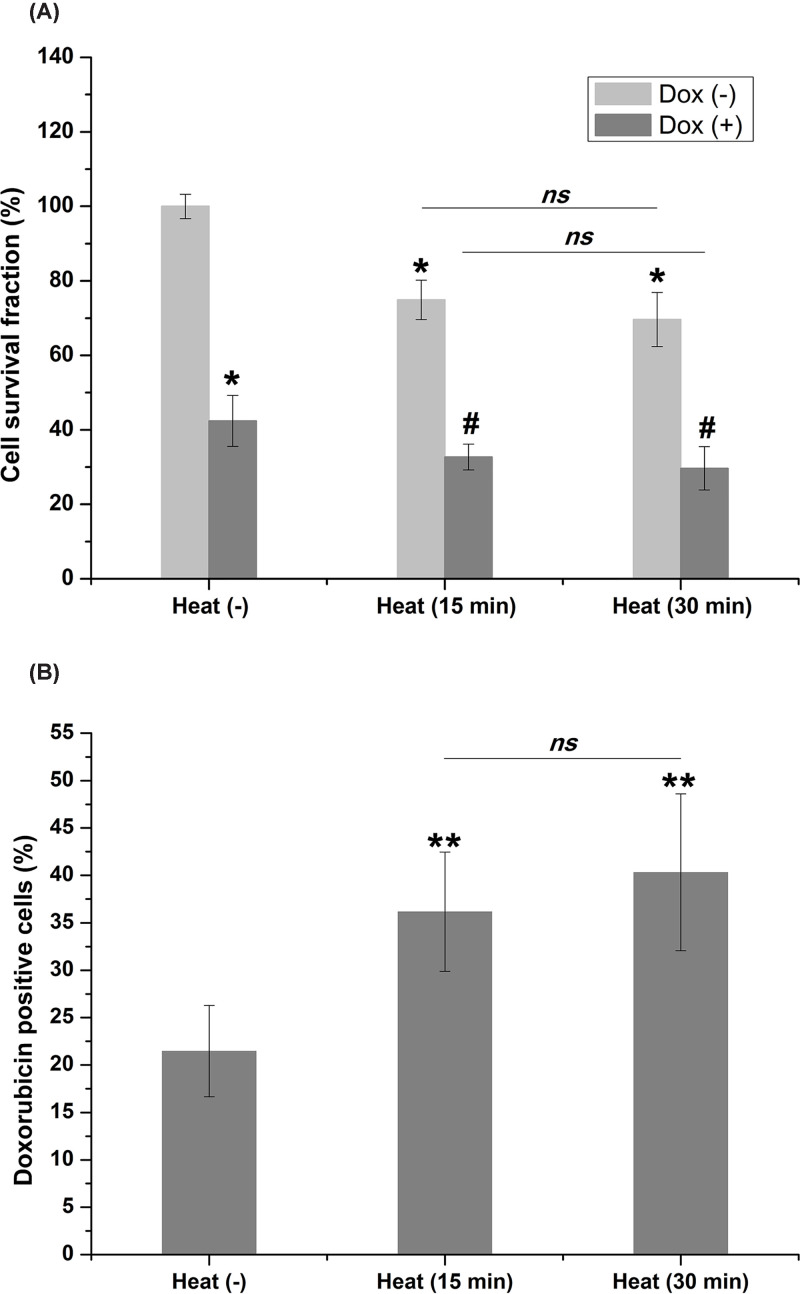
Clonogenic cell survival assay and intracellular uptake of doxorubicin (**A**) Treatment with sublethal hyperthermia or doxorubicin alone significantly influenced the cell survival (*P<0.05 vs. control). Co-treatment of sublethal hyperthermia and doxorubicin synergically inhibited cell survival (#*P*<0.05 vs. cells treated with doxorubicin alone). The difference in cell survival between cells exposed to hyperthermia (alone or in combination with doxorubicin) for 15 and 30 min was not significant (*ns*, not significant). (**B**) The percentage of doxorubicin-positive cells increased significantly following sublethal hyperthermia treatment (**P<0.01 vs. control). The difference of doxorubicin-positive cells between cells exposed to hyperthermia for 15 and 30 min was insignificant.

To evaluate the long-term effects of sublethal hyperthermia alone on chronically hypoxic liver cancer cells, hypoxic HepG2 cells were cultured in a water bath at 42°C for 15 and 30 min, and cell survival was measured using a clonogenic survival assay. The results demonstrated that the cell survival significantly decreased in cells treated with sublethal hyperthermia at 42°C for 15 min as compared with those at 37°C (74.92% ± 5.31% vs. 100% ± 3.26%, *P*<0.05). Cell survival was decreased in the cells treated with heat for 30 min as compared with those for 15 min, but the difference was not significant. For cells co-treated with hyperthermia and doxorubicin, cell survival was significantly lower than that in the cells treated with hyperthermia or doxorubicin alone. However, the difference in the cell survival fraction between cells exposed to hyperthermia for 15 and 30 min in combination with doxorubicin was not significant (Figure 1A).

Numerous studies have shown that hyperthermia enhances the intracellular uptake of doxorubicin in different types of tumor cells [20,21]. In the present study, we measured doxorubicin-positive cells using a flow cytometer. The results showed that sublethal hyperthermia significantly enhanced the intracellular uptake of doxorubicin in HepG2 cells precultured under chronically hypoxic conditions (36.17% ± 6.29% for 15 min and 40.33% ± 8.27% for 30 min vs. 21.47% ± 4.82%, *P*<0.01). However, there was no significant difference between doxorubicin-positive cells exposed to hyperthermia for 15 or 30 min (Figure 1B).

### Cell cycle analysis

**Figure 2:**
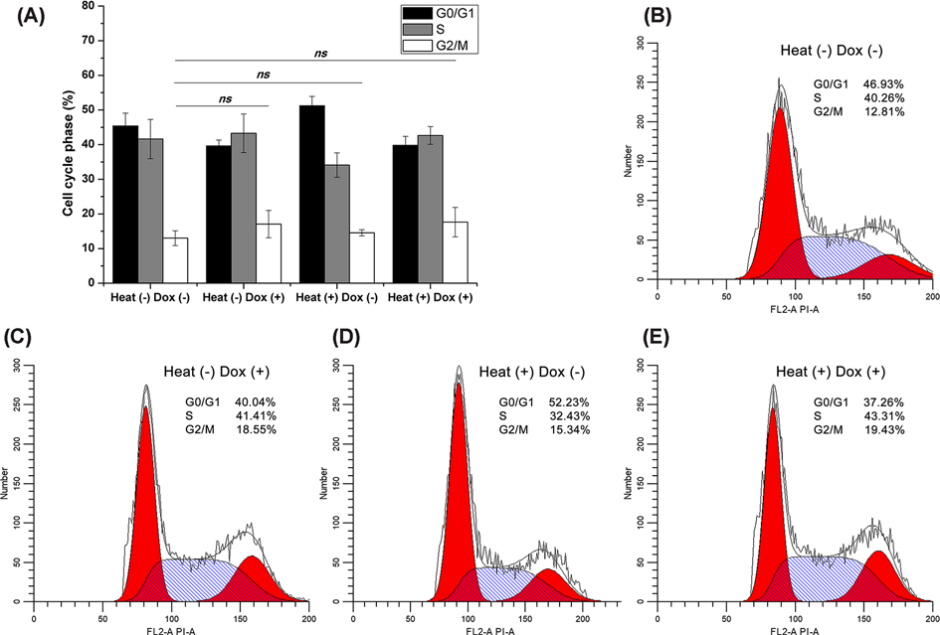
Cell cycle analysis (**A**) Treatment with sublethal hyperthermia for 15 min, doxorubicin alone, or their combination slightly influenced the cell cycle; however, the differences of cell population in various phases between groups are insignificant. (**B–E**) Representative cell cycle analysis experiment for each group.

To determine whether the inhibition of cell viability induced by hyperthermia combined with doxorubicin was attributed to cell cycle arrest, we performed cell cycle assays using flow cytometer. The results showed that treatment with sublethal hyperthermia for 15 min or doxorubicin alone had a slight influence on the cell cycle, with the G2/M population increasing from 13% ± 2.11% (control) to 14.56% ± 0.9% (hyperthermia) and 17.07% ± 3.94% (doxorubicin), but the differences were not significant. Hyperthermia in combination with doxorubicin slightly enhanced the G2/M cell cycle arrest to 17.63% ± 4.23%, but the difference was also not significant (Figure 2). Moreover, as compared with 15 min of heat exposure, treatment with hyperthermia for 30 min in combination with doxorubicin did not result in additional cell cycle alterations (data not shown). These results suggested that the cell growth inhibition induced by hyperthermia combined with doxorubicin in chronically hypoxic HepG2 cells cannot be attributed to cell cycle arrest.

### Hyperthermia combined with doxorubicin induced apoptosis via the mitochondrial pathway

**Figure 3:**
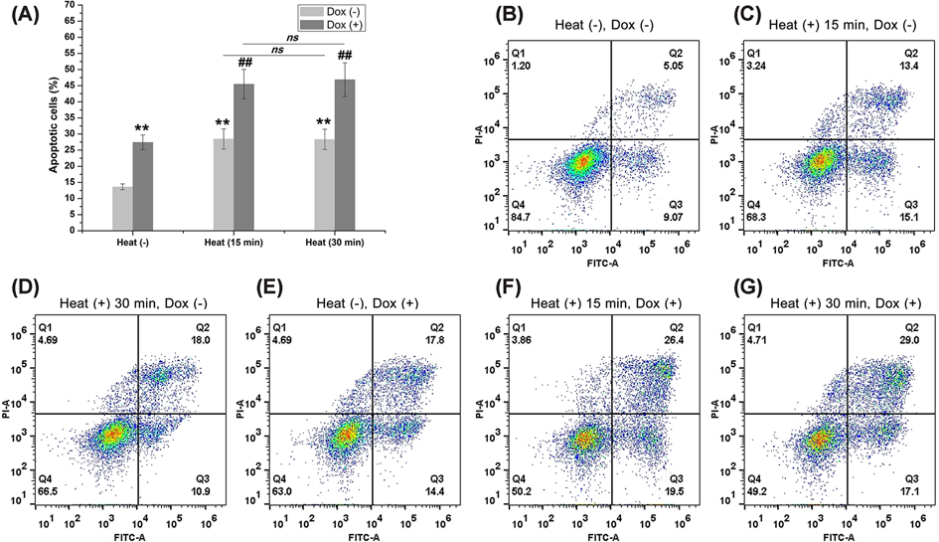
Apoptosis analysis (**A**) Treatment with sublethal hyperthermia or doxorubicin alone produced a significant increase in the apoptosis of cells (**P<0.01 vs. control). Co-treatment of sublethal hyperthermia and doxorubicin significantly increased the apoptosis of cells as compared with sublethal hyperthermia or doxorubicin alone (##*P*<0.01 vs. cells treated with doxorubicin alone). (**B–G**) Representative apoptosis analysis experiment for each group.

We then investigated whether cell death triggered by the combined treatment of hyperthermia and doxorubicin in hypoxic HepG2 cells was due to apoptosis using Annexin V/PI. Our results showed that exposure of chronically hypoxic HepG2 cells to hyperthermia for 15 min followed by doxorubicin for 24 h led to a significantly higher number of apoptotic cells (45.49% ± 4.54%) as compared with hyperthermia (28.5% ± 3.12%, *P*<0.01) or doxorubicin (27.44% ± 2.25%, *P*<0.01) alone (Figure 3). However, there was not a significantly higher number of apoptotic cells in the cells treated with hyperthermia for 30 min as compared with those treated for 15 min.

**Figure 4:**
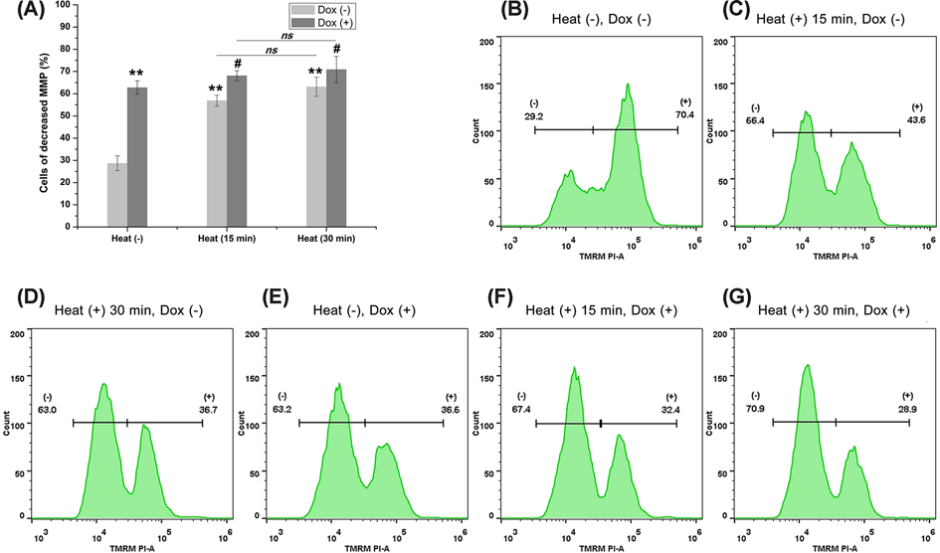
Mitochondrial membrane potential (MMP) analysis (**A**) Treatment with sublethal hyperthermia or doxorubicin alone significantly increased the percentage of cells with decreased MMP (**P<0.01 vs. control). Co-treatment of hyperthermia and doxorubicin led to a greater number of cells with alternations in MMP (#*P*<0.05 vs. cells treated with doxorubicin alone). (**B–G**) Representative MMP analysis experiment for each group.

Since increased mitochondrial permeability is an early hallmark of apoptotic cell death, we evaluated MMP to further confirm apoptosis. The results showed that treatment with hyperthermia or doxorubicin alone resulted in a marked increase in the number of cells with decreased MMP. Moreover, co-treatment of hyperthermia and doxorubicin led to a greater number of cells with alternations in MMP. Similarly, exposure of cells to hyperthermia for a relatively long time (30 min) did not result in significant MMP alterations as compared to cells exposed for a relatively short time (15 min) (Figure 4).

### Role of ROS generation and redox equilibrium

**Figure 5:**
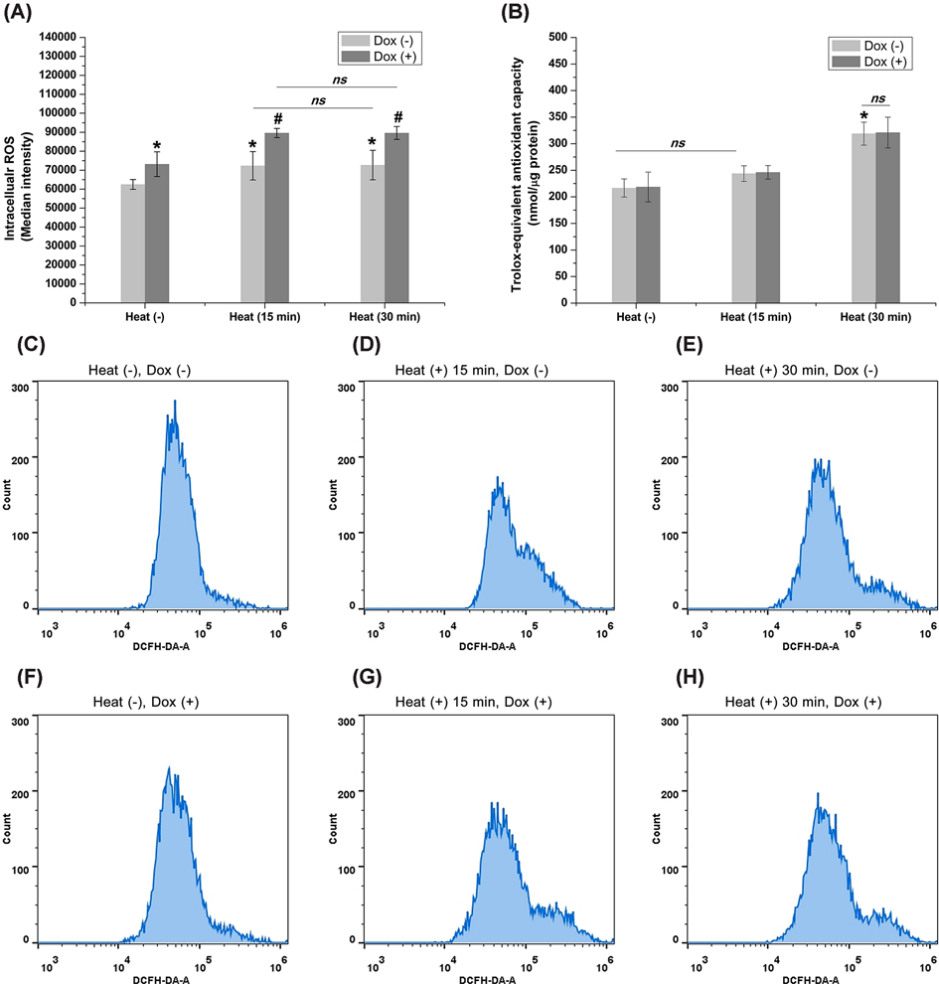
The redox equivalents were changed by heat and doxorubicin (**A**) ROS increased significantly in cells treated with hyperthermia, doxorubicin, or both (*P<0.05 vs. control; #*P*<0.05 vs. cells treated with doxorubicin alone). (**B**) Treatment with hyperthermia for a longer period of time resulted in a higher level of TAC as compared with the shorter time period (*P<0.05 vs. control). Doxorubicin did not produce additional effects on the TAC. (**C–H**) Representative ROS analysis experiment for each group.

Since ROS generation is an important early event associated with mitochondrial membrane injury and apoptosis, we then measured ROS levels in cells treated with hyperthermia and doxorubicin. After exposing chronically hypoxic HepG2 cells to hyperthermia for 15 and 30 min, ROS levels increased significantly as compared to control. However, the difference between cells exposed to hyperthermia for 15 and 30 min was not statistically significant. Cells treated with a combination of hyperthermia and doxorubicin had much higher ROS levels than those treated with hyperthermia or doxorubicin alone (Figure 5A). We also measured the TAC levels in cells treated with hyperthermia. The results showed a slight increase in the TAC level in cells exposed to hyperthermia for 15 min. However, a higher TAC level was measured in cells treated with hyperthermia for 30 min as compared with cells treated for 15 min (Figure 5B).

**Figure 6:**
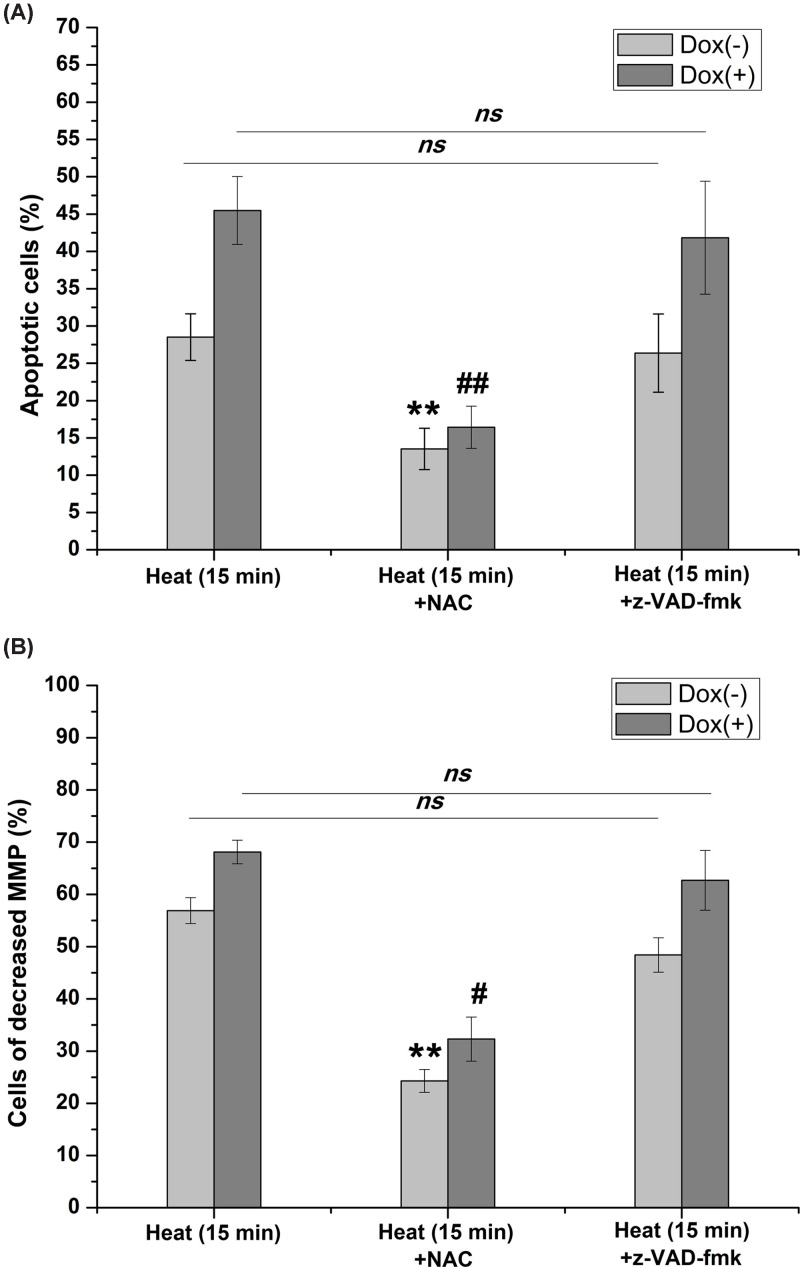
The influence of NAC and z-VAD-fmk on sublethal hyperthermia induced apoptosis and mitochondrial membrane potential dysfunction (**A**) Cellular apoptosis decreased after blocking ROS with NAC, while z-VAD-fmk produced a slight influence on cell apoptosis. (**B**) The population of cells with disrupted MMP decreased significantly after treated with NAC; similarly, the population of cells with disrupted MMP decreased slightly following z-VAD-fmk pretreatment (**P<0.01 vs*.* control; #*P*<0.05 vs. cells treated with doxorubicin alone; ##*P*<0.01 vs. cells treated with doxorubicin alone).

We then used N-acetyl cysteine (NAC), a ROS scavenger, to investigate the role of ROS in apoptosis. The results showed that the levels of apoptosis decreased significantly, which was accompanied by a decrease disrupted MMP population, in cells treated with a combination of hyperthermia and doxorubicin and NAC. We further used z-VAD-fmk, a pan caspase inhibitor, to investigate whether caspase-dependent mechanism involved in apoptosis. The results showed that z-VAD-fmk produced a slight influence on cell apoptosis and MMP (Figure 6). These results demonstrated that hyperthermia-induced apoptosis in chronic hypoxic HepG2 cells was ROS-dependent and caspase-independent.

## Discussion

Treatment of liver cancer patients with transarterial chemoembolization can lead to a tumor microenvironment characterized by chronic hypoxia. This may in turn trigger a more aggressive phenotype in the residual cancer cells [22–26]. We have previously reported that chronic hypoxia up-regulates the gene expression of NRF2/ABCB1 and PARP-1 in HepG2 cells. The NRF2/ABCB1-mediated efflux effect may lead to decreased intracellular uptake of doxorubicin, which results in chemoresistance in hypoxic liver cancer cells. In addition, PARP-1-mediated dampening of DNA damage may also play an important role in doxorubicin resistance in chronically hypoxic HepG2 cells [18].

In the present study, sublethal hyperthermia combined with doxorubicin exerted a synergistic anticancer effect *in vitro* in chronic hypoxic liver cancer cells. Our results also showed that doxorubicin-positive cells increased significantly in cells pretreated with heat at 42°C. These results are consistent with results reported in the literature where hyperthermia ranging from 41 to 47°C altered cell membrane permeability and in turn allowed uptake of chemotherapeutic agents [21,27,28]. However, this effect has not been observed in all procedures where hyperthermia was combined with chemotherapy [29]. There was no significant difference in the inhibition of cell survival in cells pretreated with heat for 30 or 15 min. Additionally, the difference in doxorubicin-positive cells between these two groups was not statistically significant. These results indicated that the synergistic effect of co-treatment may be partially explained by hyperthermia-mediated intracellular uptake of doxorubicin.

Both hyperthermia and doxorubicin have been shown to cause cell cycle arrest in a broad range of cancer cells. Cell cycle analysis in the present study showed that hyperthermia or doxorubicin alone or a combination of both had a slight, direct effect on cell cycle distribution in chronically hypoxic HepG2 cells. This finding is inconsistent with those reported by Fatfat et al. and Lim et al., where both hyperthermia and doxorubicin dramatically affected the cell cycle distribution *in vitro* in liver cancer cells [30,31]. This inconsistency may be explained by the dose of hyperthermia or doxorubicin applied in the present study, which differed from what was reported previously [32,33]. In addition, the same dose of inducer may have a different effect on cells in various states.

Many studies have shown that the biological effect of hyperthermia is correlated with chemically reactive molecules containing oxygen, also known as ROS [34,35]. In the present study, our results showed that both hyperthermia and doxorubicin induced the generation of ROS, resulting in a decrease in MMP and apoptosis. These results suggested that inhibition of ROS reversed hyperthermia- and doxorubicin-induced apoptosis. However, exposure of cells to sublethal hyperthermia for a relatively long time (30 min) did not result in a higher ROS level. This suggested that the generation of ROS following sublethal hyperthermia was not time-dependent. Interestingly, our results also showed that the 30-min exposure of cells to hyperthermia led to a higher TAC level as compared with cells exposed for 15 min. A high TAC may aid tumor cells in maintaining a high-redox equilibrium for survival under heat stress, thus triggering thermo-tolerance in cancer cells [36,37]. These results hint that a longer time period of thermal ablation may theoretically lead to increased necrosis in the lethal hyperthermia zone. Alternatively, it may also lead to thermo-tolerance in the sublethal hyperthermia zone through redox equilibrium. Thus, further studies are needed to investigate the potential dual effects of sublethal hyperthermia during thermal ablation. Chen et al. reported that downstream effectors of NRF2, ABCC1, contribute directly to acquired resistance and survival in several types of tumor cells through the mediation of redox equilibrium [38]. Jeon et al. investigated gene expression profiles of modulated electro-hyperthermia (modulated electro-hyperthermia) mEHT-treated HepG2 cells [39]. These results demonstrated that mEHT inhibited HCC cell growth through a subset of molecular changes. Whether sublethal hyperthermia produces similar effects on global gene expression profiles, which in turn influences redox equilibrium and results in apoptosis in chronic hypoxic liver cancer cells, needs further investigation.

There are a couple of limitations to this study. First, the conditions of hyperthermia used in the present study may not fully mimic the actual status of the periablation zone during thermal ablation, where a gradual decrease in temperature often occurs. Second, our results showed that sublethal hyperthermia induced high levels of TAC, which may trigger thermal tolerance in hypoxic liver cancer cells. However, the underlying mechanism remains to be determined. Thus, further studies are needed to investigate the effects of a wider range of sublethal thermal heating doses on more hepatic cancer cell lines and animal models, as well as the detailed underlying molecular mechanisms.

In summary, this study showed that sublethal hyperthermia enhanced doxorubicin-induced cytotoxicity in chronically hypoxic HepG2 cells through an increased intracellular uptake of doxorubicin, generation of ROS, and mitochondrial membrane injury. In addition, a relatively long exposure time to sublethal hyperthermia induced a high TAC, which may trigger thermo-tolerance in chronically hypoxic liver cancer cells. These findings suggest a new strategy for improving efficacy and overcoming potential thermo-tolerance in tumor cells in the periablation zone during thermal ablation in HCC patients.

## Supplementary Material

Supplementary Figure S1

## Data Availability

The datasets generated during and/or analysed during the current study are available from the corresponding author on reasonable request.

## Abbreviations

Dox: doxorubicin
HCC: hepatocellular carcinoma
mEHT: modulated electro-hyperthermia
MMP: mitochondrial membrane potential
MWA: microwave ablation
NAC: N-acetyl cysteine
RFA: radiofrequency ablation
ROS: reactive oxygen species
TAC: total antioxidant capacity
TACE: transarterial chemoembolization
TAE: transarterial embolization
