# Supplementary material for: Sublethal hyperthermia enhances anticancer activity of doxorubicin in chronically hypoxic HepG2 cells through ROS-dependent mechanism
Source: Biosci Rep. 2021 Jun 11;41(6):BSR20210442. doi: 10.1042/BSR20210442 (PMC8200658; doi:10.1042/BSR20210442)
Supplement: Supplementary Figure S1 [file BSR-2021-0442_supp.pdf]

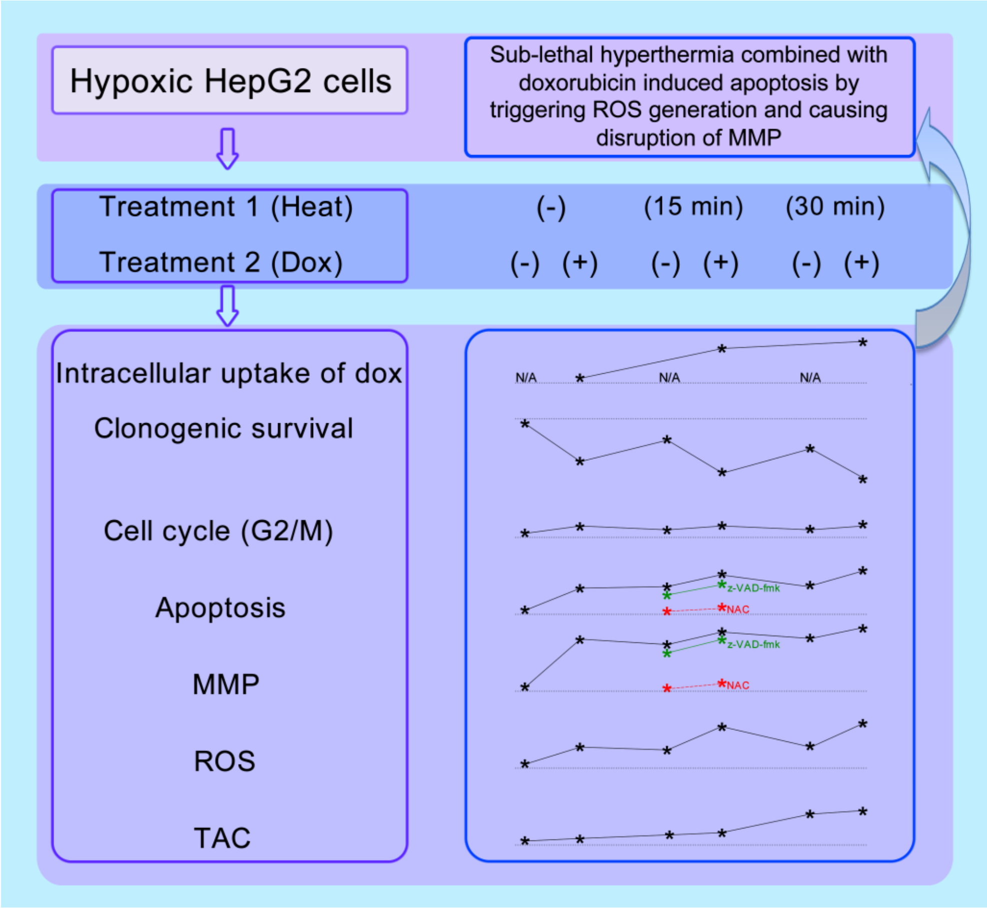

Supplementary figure 1. Graphic abstract summarizing all the experimental steps and the results.
